# Hyperwrinkling of the nuclear lamina is associated with attenuated mechanosensitivity in giant nucleated cancer cells

**DOI:** 10.1126/sciadv.aed1645

**Published:** 2026-07-17

**Authors:** Samere Abolghasemzade, Ryan Blanchard, Chance French, Christina R. Dollahon, Harrison Pham, Aidan Atkins, Richard B. Dickinson, Tanmay P. Lele

**Affiliations:** ^1^ Department of Biomedical Engineering, Texas A&M University, College Station, TX, USA.; ^2^ Department of Chemical Engineering, University of Florida, Gainesville, FL, USA.; ^3^ Artie McFerrin Department of Chemical Engineering, Texas A&M University, College Station, TX, USA.; ^4^ Department of Translational Medical Sciences, Texas A&M University, Houston, TX, USA.; ^5^ Texas A&M University School of Engineering Medicine, 1020 Holcombe Blvd, Houston, TX 77030.

## Abstract

Polyploid giant cancer cells (PGCCs) are chemoresistant tumor cells associated with poor patient outcomes. PGCCs in tissue are identified by their extremely large sizes and the presence of massive or several nuclei. Although nuclear dysmorphia is also a common characteristic of tumor cells, little is known about the shape of nuclei in PGCCs. We show that the nuclear lamina in giant nucleated PGCCs is highly wrinkled across diverse cancer patient tissues. We investigated the cause of this hyperwrinkling in ovarian PGCCs in vitro. Laminar hyperwrinkling in PGCCs was independent of cell shape or cytoskeletal forces. Instead, measurements combined with computational modeling show that laminar hyperwrinkling is an intrinsic property of PGCCs, arising from a disproportionate amount of laminar excess area. PGCCs also displayed attenuated mechanosensitivity of cell spreading and YAP nuclear localization compared with control cells. Thus, laminar hyperwrinkling may disrupt PGCC mechanobiological pathways, slowing their proliferation.

## Teaser

Nuclear lamina hyperwrinkling in giant cancer cells arises from excess laminar area and correlates with impaired mechanosensing.

## INTRODUCTION

Nuclear atypia has long been a defining histopathological feature of cancer progression (*1*, *2*). Recently, polyploid giant cancer cells (PGCCs), which arise in many tumor types under reproductive stress such as chemotherapy or hypoxia, have been identified in diverse cancers (*3*–*8*). These cells contain either one abnormally large nucleus or several nuclei and form through failed mitosis and accumulation of excess DNA (*9*–*11*). Giant nucleated PGCCs (GN-PGCCs), which initially appear to not be able to divide, can produce daughter cells through amitotic budding, contributing to aggressive metastasis and treatment resistance (*3*, *10*, *12*, *13*). Consistent with this, the presence of PGCCs in patient biopsies is a prognostic marker for poor outcomes (*5*, *14*–*16*).

GN-PGCCs are traditionally identified in hematoxylin and eosin (H&E)–stained tissues based on their large nuclear size (*3*, *17*). Nuclear shape irregularities are commonly observed in cancer (*1*), yet the extent to which GN-PGCC nuclei adopt abnormal morphologies is unknown. This may be because it is difficult to discern micrometer-scale undulations in the nuclear surface in routine H&E histology (*18*–*23*). Nuclear lamins, which form a 15-nm-thick meshwork beneath the inner nuclear membrane (*24*, *25*), mechanically determine nuclear shapes in some mammalian cell types (*1*, *26*–*33*). Thus, immunofluorescence imaging of nuclear lamins offers the opportunity to directly visualize the nuclear surface and its fine-scale deformations.

Here, we applied this approach to examine nuclear morphology in GN-PGCCs. GN-PGCC nuclei tended to exhibit hyperwrinkling of the nuclear lamina across diverse patient tumor tissues and in an in vitro model of ovarian GN-PGCCs. These wrinkles could not be explained by cell rounding or mechanical indentation by cytoskeletal structures, which suggest that hyperwrinkling is an intrinsic feature of GN-PGCC nuclei. We have recently shown that unwrinkling of the nuclear lamina correlates with the mechanosensitive nuclear import of the transcriptional coregulator yes-associated protein (YAP) (*32*). Consistent with these prior studies, here, we demonstrate that GN-PGCCs fail to smooth their nuclei and correspondingly lack mechanosensitive YAP nuclear localization on stiff extracellular matrices (ECMs).

## RESULTS

### Giant nuclei in GN-PGCCs exhibit a highly wrinkled lamina in cancer patient tissues

**Fig. 1.**
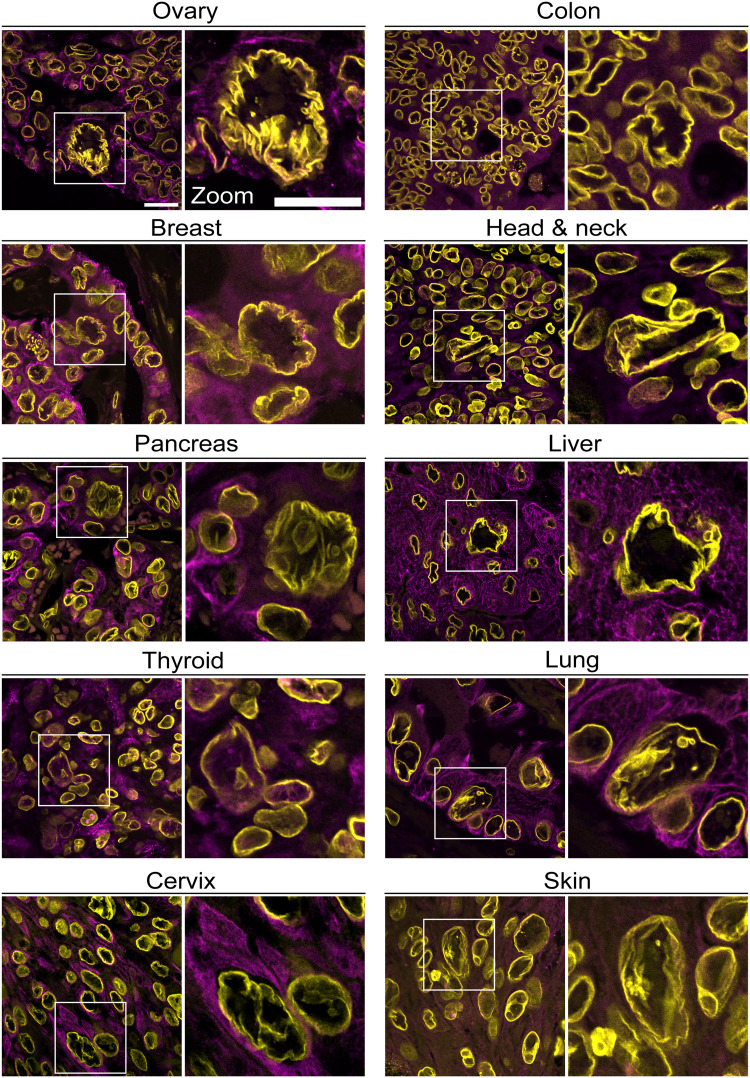
Giant nuclei in GN-PGCCs are highly wrinkled in diverse cancer patient tissues. Confocal images at 60× magnification of giant nuclei in FFPE ovarian, breast, pancreatic, thyroid, cervical, colon, head and neck, liver, lung, and skin cancer tissues stained for lamin B1 (yellow) and pan-cytokeratin (magenta). Larger images show surrounding nongiant cancer cells, with white boxes denoting zoomed regions with PGCCs. Regions were selected by the presence of a nucleus at least three times as large as surrounding epithelial cells. All scale bars are 20 μm.

Previously, we showed that epithelial nuclei in cancer tissues exhibit greater laminar wrinkling than those in control tissue (*19*). Motivated by these studies, here, we asked if giant nuclei in GN-PGCCs are also wrinkled. We performed high-resolution confocal imaging of formalin-fixed paraffin-embedded (FFPE) human tissue microarrays immunostained for lamin B1 (to label the lamina) and pan-cytokeratin (to identify epithelial cells). We identified the presence of giant nuclei in the collected images across 10 different cancer types (Fig. 1 and fig. S1); giant nuclei were defined as those nuclei that were at least three times larger than neighboring cancer nuclei. Cells with these giant nuclei, i.e., GN-PGCCs, were found to be extremely rare in control (cancer adjacent) tissues but were consistently observed in tumors of all grades (Tables 1 and 2), present in 3 to 25% of patient tumors depending on the cancer type. Across all tissue types (ovarian, breast, cervical, colon, head and neck, skin, thyroid, lung, pancreas, and liver cancer), both nongiant and giant nuclei tended to be wrinkled, consistent with our prior report that extreme laminar wrinkling is a morphological marker of cancer (*19*). Notably, in most cases, the giant nuclei exhibited more severe, “hyperwrinkled” laminae compared with surrounding nongiant cancer nuclei. Using our previously trained deep learning model (*19*), we sorted the nuclei into four categories of nuclear wrinkling (fig. S2A). Fewer smooth nuclei were present in the PGCCs, and a higher proportion of extreme wrinkling (classes 3 and 4) was observed in PGCCs compared to non-PGCC carcinoma cells, supporting the presence of hyperwrinkling in PGCC populations (fig. S2B).

**Table 1.** Prevalence of giant nuclei in cancer tissues. The number of GN-PGCC-positive patients and images, represented as positive (total), counted from lamin B1 and pan-cytokeratin–immunostained sections of 10 different carcinomas and associated normal and/or cancer-adjacent tissues. GN-PGCC–positive images and patients were defined as those with at least one in-focus epithelial nucleus which was at least 3× larger than some surrounding epithelial nuclei. Cells were considered to be epithelial if they expressed pan-cytokeratin. The bold font indicates that the “Pooled cancer” numbers are the sum of all the cancer numbers (excluding cancer adjacent/normal) for each tissue type.

Ovary*	Normal/normal adjacent	Mucinous	Serous	Low-grade serous	High-grade serous		Pooled cancer
PGCC-positive patients	0 (3)	5 (35)	4 (23)	0 (5)	1 (26)		
PGCC-positive images	0 (9)	5 (78)	5 (62)	0 (9)	1 (49)		
Ovary, cont.	**Grade 1**	**Grade 2**	**Grade 3**	**Ovary metastasis**			
PGCC-positive patients	0 (3)	0 (8)	2 (17)	5 (12)			**17 (127)**
PGCC-positive images	0 (5)	0 (16)	3 (31)	8 (34)			**22 (284)**
Breast†	**Cancer adjacent** ‡	**IDC**	**Grade 1**	**Grade 2**	**Grade 3**		
PGCC-positive patients	1 (32)	1 (16)	0 (10)	6 (39)	1 (12)		**8 (77)**
PGCC-positive images	1 (57)	1 (31)	0 (12)	6 (60)	1 (18)		**8 (122)**
Pancreas	**Normal**	**Grade 1**	**Grade 2**	**Grade 3**			
PGCC-positive patients	1 (8)	0 (5)	2 (19)	5 (11)			**7 (35)**
PGCC-positive images	1 (8)	0 (5)	2 (19)	5 (11)			**7 (35)**
Thyroid	**Normal**	**Cancer**					
PGCC-positive patients	0 (10)	2 (64)					
PGCC-positive images	0 (10)	2 (64)					
Cervix	**Normal**	**Grade 1**	**Grade 2**	**Grade 3**			
PGCC-positive patients	0 (6)	2 (13)	5 (22)	1 (5)			**8 (40)**
PGCC-positive images	0 (6)	2 (13)	5 (22)	1 (5)			**8 (40)**
Colon	**Normal** §	**Grade 1**	**Grade 2**	**Grade 3**			
PGCC-positive patients	1 (12)	3 (14)	6 (45)	1 (17)			**10 (76)**
PGCC-positive images	2 (28)	3 (34)	9 (118)	1 (38)			**13 (190)**
Head and neck¶	**Normal/normal adjacent**	**Grade 1**	**Grade 2**	**Grade 3**			
PGCC-positive patients	0 (6)	2 (9)	1 (6)	2 (6)			**5 (20)**
PGCC-positive images	0 (33)	4 (50)	1 (28)	2 (26)			**7 (122)**
Liver	**Normal**	**Grade 2**	**Grade 2–3**	**Grade 3**			
PGCC-positive patients	0 (8)	1 (15)	2 (6)	2 (13)			**5 (34)**
PGCC-positive images	0 (8)	1 (15)	2 (6)	2 (13)			**5 (34)**
Lung	**Normal**	**Grade 2**	**Grade 2–3**	**Grade 3**	**Small CC**	**Large CC**	
PGCC-positive patients	0 (9)	4 (30)	1 (14)	3 (34)	0 (6)	0 (3)	**8 (87)**
PGCC-positive images	0 (19)	4 (30)	1 (14)	3 (34)	0 (6)	0 (3)	**8 (87)**
Skin#	**Cancer adjacent**	**BCC**	**Grade 1**	**Grade 2**	**Grade 3**		
PGCC-positive patients	0 (10)	0 (14)	2 (22)	4 (13)	1 (6)		**7 (55)**
PGCC-positive images	0 (20)	0 (28)	2 (44)	5 (26)	1 (12)		**8 (110)**

* Two patients are represented in serous and low-grade serous, one patient in grades 1 and 2, and three patients in grades 2 and 3.

† Eighteen cases contained both cancer and cancer adjacent tissue in this analysis.

‡ Because of large nuclear size variation in cancer adjacent tissue, nuclei needed to be 3× larger than most to qualify as PGCCs.

§ Because of large nuclear size variation in normal colon tissue, nuclei needed to be 3× larger than most to qualify as PGCCs.

¶ One patient is represented in grades 1 and 2, and one patient in grades 2 and 3.

# Ten cases contained both cancer and cancer adjacent tissue in this analysis.

**Table 2.** Abbreviations and cancer types.

Ovary	Mucinous adenocarcinoma (papillary, NOS); serous adenocarcinoma (papillary, NOS); low-grade, high-grade serous carcinoma; grades 1 to 3 adenocarcinoma (endometrioid, NOS); metastatic [lymph node metastasis (clear cell carcinoma); serous adenocarcinoma (papillary and NOS)]
Breast	IDC: intraductal carcinoma [pure DCIS (ductal carcinoma in situ) and intraductal with invasive cancer elsewhere]; invasive ductal carcinoma; medullary carcinoma
Pancreas	Pancreatic ductal adenocarcinoma
Thyroid	Follicular, papillary, and undifferentiated carcinoma
Cervix	Squamous cell carcinoma, not otherwise specified (NOS); adenocarcinoma (HPV-associated, gastric type, clear cell type, endometrioid, NOS)
Colon	Adenocarcinoma (mucinous, papillary, with neuroendocrine differentiation, NOS); signet ring cell carcinoma
Head and neck	Squamous cell carcinoma
Liver	Hepatocellular carcinoma
Lung	Squamous cell carcinoma, adenocarcinoma (mucinous, NOS); small CC: small-cell carcinoma; large CC: large-cell carcinoma
Skin	BCC: basal cell carcinoma (fibrous epithelial, nodular, small nodular, squamous cell, sclerosis, pigmented, invasive, and NOS types); squamous cell carcinoma

Although H&E staining is the gold standard for cancer diagnosis in tissues based on nuclear shape, we and others have reported that H&E images of diverse patient tissues fail to capture the spatial detail of nuclei necessary to visualize wrinkling compared to nuclear membrane protein immunostaining (*19*, *23*). Consistent with these results, H&E-stained images of the same giant nuclei as shown above did not provide evidence of nuclear hyperwrinkling (fig. S3). Collectively, these results suggest that laminar hyperwrinkling, in addition to nuclear enlargement, may be a defining morphological feature of GN-PGCCs.

### Laminar wrinkling does not reflect a lack of nuclear flattening in PGCCs

In spread, cultured epithelial cells which have flattened nuclei, the nuclear lamina is typically smooth; however, it can develop wrinkles rapidly when the cell shape is rounded up (*34*). The wrinkling develops in the rounded shape as a consequence of a geometric principle: When a flattened smooth shape is forced toward a more spherical shape without changing its volume or surface area, wrinkles will develop in the surface, because a sphere has the smallest surface area for its volume.

**Fig. 2.**
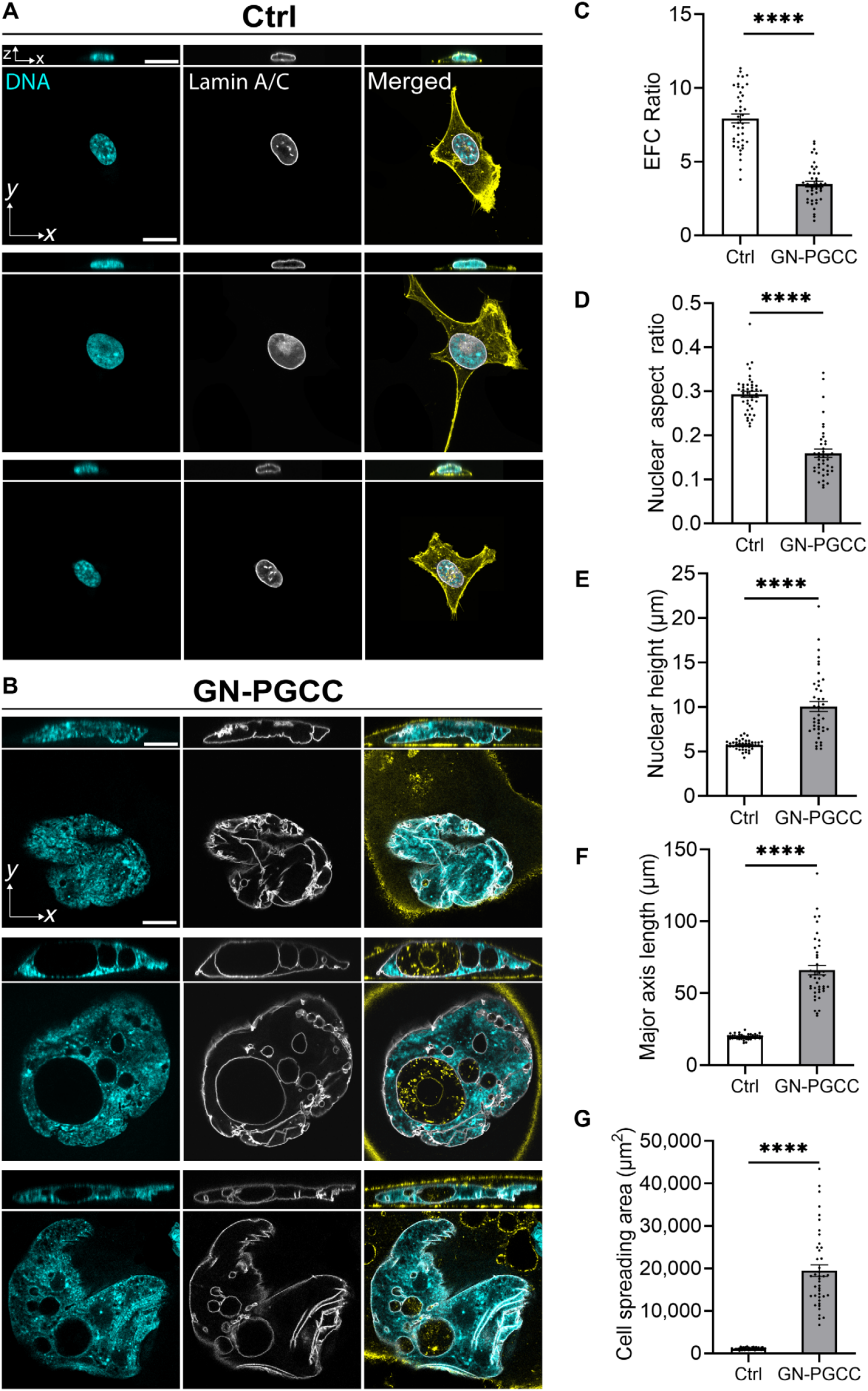
Laminar wrinkling does not reflect a lack of nuclear flattening in GN-PGCCs. Confocal images of (**A**) HEY control cells and (**B**) GN-PGCC expressing GFP–Lamin A (gray) cultured on glass. Cells were stained for DNA (cyan), and F-actin (yellow). All scale bars are 20 μm. Quantification and comparison of (**C**) EFC ratio, (**D**) nuclear aspect ratio (height/long axis length), (**E**) nuclear height, (**F**) major axis length, and (**G**) cell spreading area between control cells and GN-PGCCs. Statistically significant differences were determined by Student’s *t* test, ***P < 0.001, ****P < 0.0001, *n* = 42, 42 for control cells and GN-PGCCs, respectively. Quantitative data were collected from three independent biological replicates.

To test whether laminar wrinkles in GN-PGCCs are due to a lack of nuclear flattening, we examined laminar morphology in cultured PGCCs in vitro. GN-PGCCs were generated by treating HEY human ovarian cancer cells stably expressing GFP–lamin A (to visualize the nuclear lamina) with 10 nM docetaxel for 3 days, followed by 1 day of recovery in fresh medium, and a second 3-day exposure to 10 nM docetaxel (*35*, *36*). Control cells and GN-PGCCs were next cultured in fresh medium (without the drug) on fibronectin-coated glass substrates and imaged with high-resolution confocal microscopy. As seen in Fig. 2A, control Hey cells featured a smooth nuclear lamina with a corresponding flattened nuclear cross section in the *x*-*z* plane and a well-organized F-actin cytoskeleton. GN-PGCCs also exhibited highly flattened nuclear cross-sections in the *x*-*z* plane (Fig. 2B). However, in the *x*-*y* plane, these nuclei were notably irregular in contour, with extensive hyperwrinkling of the lamina. These wrinkles were so extensive that they could not be smoothened even by hypo-osmotic shock (fig. S4A), which we have previously shown can unfold nuclear wrinkles through an increase in nuclear volume (*32*). In addition, large intranuclear cavities in some GN-PGCCs were occasionally observed, which in some cases contained actin filaments or lipid droplets, consistent with previous reports of lipid accumulation in PGCCs (Fig. 2B and fig. S4) (*37*, *38*).

To assess these observations at the population level, we quantified nuclear contour irregularity using elliptical Fourier analysis (*19*, *39*–*41*). This approach computes the elliptical Fourier coefficient (EFC) ratio, which serves as an inverse measure of contour irregularity. For each nucleus, the mean EFC ratio averaged over three *z*-planes was computed as previously described (*32*). GN-PGCC nuclei had a significantly lower EFC ratio than the control nuclei, consistent with their wrinkling appearance (Fig. 2C). Yet, vertical aspect ratios of the nucleus, which quantify the degree of nuclear flattening, were significantly lower in GN-PGCCs compared to control cells (Fig. 2D), indicating that GN-PGCC nuclei were more flattened than control nuclei. Nuclear height in GN-PGCCs was roughly twice that of controls (Fig. 2E), while the major axis of the GN-PGCC nucleus was nearly threefold greater (Fig. 2F). In addition, GN-PGCC cells were also highly spread compared to control (Fig. 2G). Together, these findings show that hyperwrinkling in GN-PGCCs cannot be explained by a failure to spread or flatten their nuclei.

### Nuclear wrinkling in GN-PGCCs is not induced by cytoskeletal forces

**Fig. 3.**
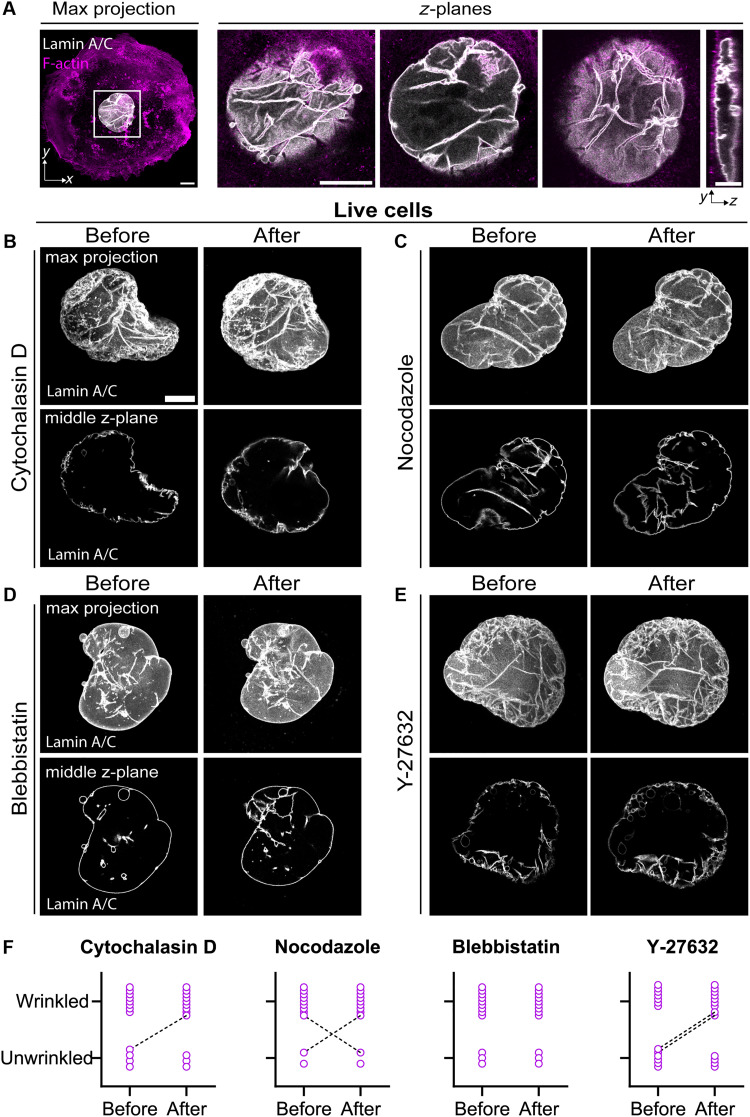
Cytoskeletal and actomyosin disruption does not affect nuclear laminar wrinkling in GN-PGCCs. (**A**) Super-resolution micrograph of fixed GFP–lamin A GN-PGCC stained for F-actin. Max projection, middle *z*-plane, and *y*-*z* view are displayed. All scale bars are 20 μm, except for YZ projection, which is 10 μm. Confocal max projection and middle *z*-plane images of live GFP–lamin A GN-PGCCs before and after treatment with (**B**) cytochalasin D, (**C**) nocodazole, (**D**) blebbistatin, and (**E**) Y-27632. Scale bars are 20 μm. (**F**) Number of wrinkled and unwrinkled nuclei before and after each drug treatment. Dashed lines depict the rare cases when nuclear state was altered by drug treatment. A lack of a dashed line indicates no change in wrinkling state. *n* = 11, 12, 12, and 13 for GN-PGCCs treated with nocodazole, cytochalasin D, blebbistatin, and Y-27632, respectively. Quantitative data were collected from three independent biological replicates.

Another possible explanation for the wrinkles found in giant nuclei is that cytoskeletal structures mechanically indent the nuclear surface (*42*, *43*). We performed super-resolution imaging of the F-actin cytoskeleton and the lamina to test if wrinkles colocalized with nuclear-indenting F-actin fibers. Super-resolution imaging successfully resolved the apparent single laminar lines in the confocal images as double lines in that plane, suggestive of surfaces on either side of a fold (Fig. 3A). However, the colocalization of F-actin fibers with these wrinkles was inconsistent, with F-actin present in some wrinkles but not others. This suggests that F-actin may not be a driver of wrinkling in PGCCs.

We next perturbed cytoskeletal structures pharmacologically. GN-PGCCs were treated with cytochalasin D and nocodazole to depolymerize actin and microtubules, respectively, and blebbistatin or Y27632 to inhibit nonmuscle myosin II and Rho kinase, respectively. Live-cell imaging of green fluorescent protein (GFP)–lamin A expressing GN-PGCCs revealed no discernible changes in laminar wrinkling or gross shape under any of these conditions (Fig. 3, B to F). Fixed imaging confirmed effective cytoskeletal disruption with clear depolymerization of F-actin and microtubules (fig. S5, A and B). Quantification of the EFC ratio in the population of fixed cells likewise showed no significant differences in contour irregularities across treatments (fig. S5C). We confirmed that cytoskeletal or contractile disruption had no effect on the frequency of wrinkling in WT GN-PGCCs (fig. S5D); also, WT GN-PGCCs contained approximately the same proportion of wrinkled nuclei as the GFP–lamin A expressing GN-PGCCs (75.3% versus 77.7%). Collectively, these results suggest that laminar wrinkling in GN-PGCCs is not caused by mechanical indentation of the nuclear surface by cytoskeletal structures.

### Hyperwrinkling is an intrinsic feature of GN-PGCC nuclei

**Fig. 4.**
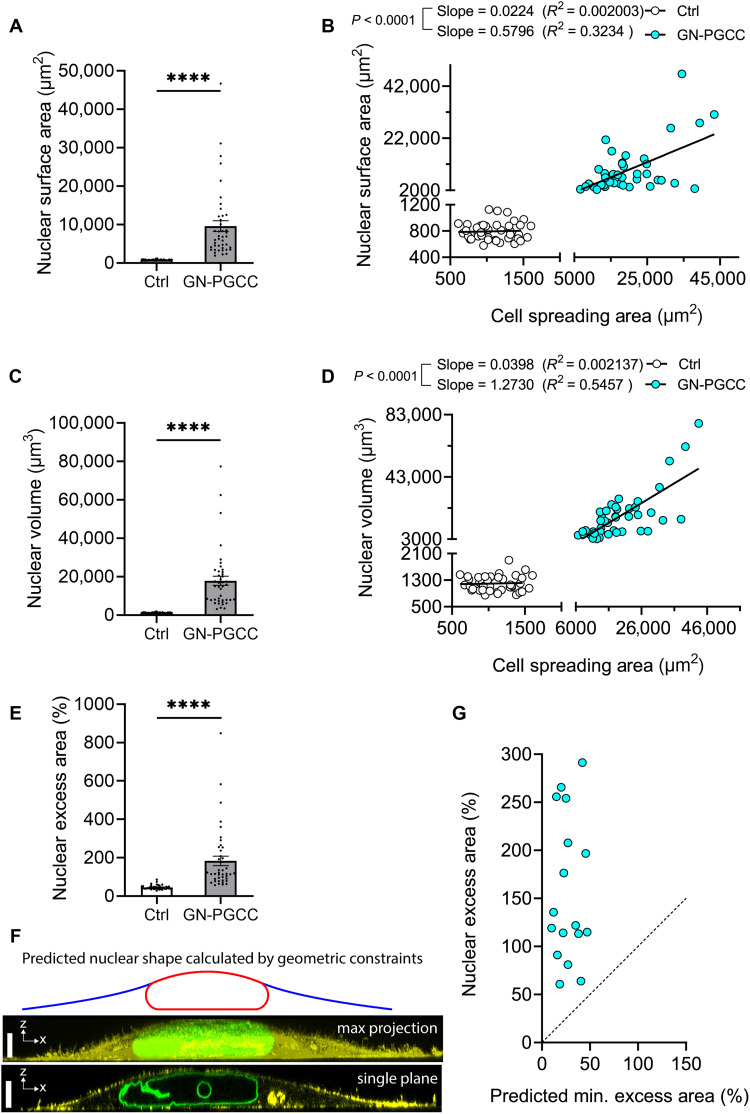
Nuclear morphometry of giant nuclei reveals the presence of large excess area. Quantification and comparison of (**A**) nuclear surface area, (**B**) nuclear surface area correlated to cell spreading area, (**C**) nuclear volume, (**D**) nuclear volume correlated to cell spreading, and (**E**) nuclear excess area between control cells and GN-PGCCs. (**F**) Calculated shapes of cell (blue) and nucleus (red) for a smooth, constrained nucleus under the cell cortex. Scale bar is 5 μm. (**G**) Comparison of nuclear excess area to predicted minimum excess area for the same nucleus. Slopes and *R*^2^ values of linear fitting and *P* values for slope comparisons are shown on the graphs. Statistically significant differences were determined by Student’s *t* test, ***P < 0.001, ****P < 0.0001, *n* = 42, 42 for control cells and GN-PGCCs, respectively. Quantitative data were collected from three independent biological replicates.

To further examine the cause of hyperwrinkling in GN-PGCCs, we asked whether it could be explained by an excessive nuclear surface area for its volume. Morphometric analysis showed that nuclear surface area in GN-PGCCs was significantly larger than nongiant control nuclei and unlike in control cells, scaled with the cell spreading area (Fig. 4, A and B). Likewise, nuclear volume was significantly larger in GN-PGCCs than control nuclei and similarly correlated with spreading (Fig. 4, C and D). Despite this increased size, GN-PGCC nuclei remained highly wrinkled, suggesting the presence of unusually high surface area. To test this directly, we quantified excess area, defined as the laminar surface area in excess of the area of a sphere of equal volume (*44*). By definition, a smooth sphere has zero excess area and no wrinkles, while an approximately spherical shape with high excess area will display high levels of wrinkling. Notably, the excess area was nearly four times higher in GN-PGCCs than in control cells (Fig. 4E).

We next applied our previously developed geometric model to computationally calculate the minimum excess area of the nucleus for a given volume and axisymmetric cell shape (*26*, *32*, *44*). For direct comparison with experimental data, we selected axisymmetric cells from our dataset and input their nuclear volume, cell height (used to calculate the volume of the axisymmetric cell) and cell spreading area into the model. The model was used to calculate the nuclear shape with the minimum nuclear surface area, i.e., with a smooth surface (Fig. 4F). The calculated minimum surface area was used, along with the volume, to compute the minimum excess area for the predicted nuclear shape. Comparison of the model-calculated excess area with the experimentally measured excess area showed that the datapoints consistently lay above the 45° line (Fig. 4G), with the experimentally measured excess area far exceeding the computationally calculated excess area. These findings suggest that GN-PGCC nuclei have far more excess surface area than needed to establish a flattened smooth nuclear shape. As a result, even flattened, well spread cells are unable to unfold the large amounts of excess area in these nuclei. Thus, hyperwrinkling is an intrinsic feature of GN-PGCC nuclei.

### GN-PGCCs are less mechanosensitive than parental cells

A hallmark of tumor progression, including ovarian tumors, is a gradual mechanical stiffening of the ECM (*45*–*51*). Cancer cells are typically mechanosensitive: They respond to matrix stiffening through increased adhesion and spreading, and the concomitant nuclear localization of the transcriptional coregulator YAP (*32*) where it promotes proliferation (*52*–*55*). How matrix stiffening promotes YAP nuclear localization is still not fully understood (*55*). We have recently reported that the presence of an unwrinkled nuclear lamina in spread cells on stiff hydrogels correlates with increased YAP nuclear localization (*32*) across several cancer cell types. An unwrinkling of the lamina results in an in-plane tension that depends on lamin A/C, and depletion of lamin A/C disrupts the sensitivity of YAP nuclear localization to cell spreading. Given the laminar hyperwrinkling of GN-PGCCs, whether GN-PGCCs can unwrinkle their lamina on stiff hydrogels relative to soft hydrogels and whether unwrinkling in GN-PGCCs correlates with YAP nuclear localization are unclear.

**Fig. 5.**
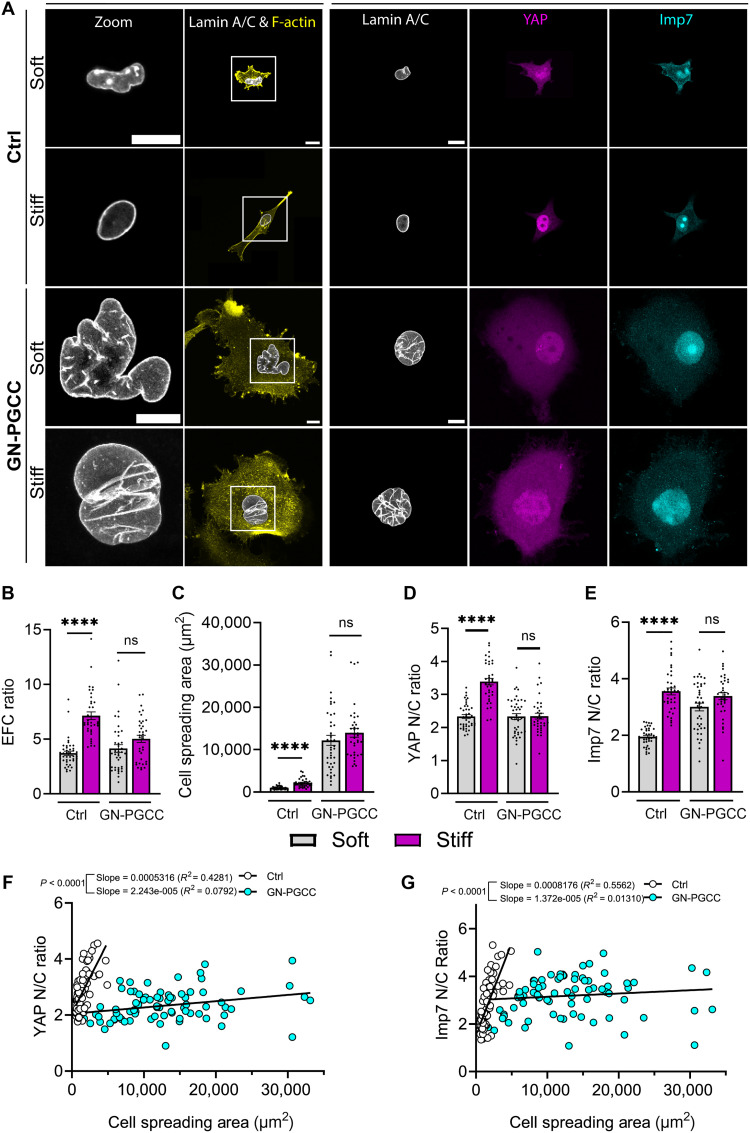
GN-PGCCs have reduced mechanosensitivity compared to parental cells. (**A**) Confocal images of HEY control cells and GN-PGCCs expressing GFP–Lamin A (gray) cultured on soft (1 kPa) and stiff (308 kPa) polyacrylamide hydrogels. Cells were stained for F-actin (yellow), YAP (magenta), and importin 7 (cyan). All scale bars are 20 μm. Quantification and comparison of (**B**) EFC ratio, (**C**) cell spreading area, (**D**) YAP nuclear to cytoplasmic ratio, (**E**) importin 7 nuclear to cytoplasmic ratio, (**F**) YAP nuclear to cytoplasmic ratio correlated to cell spreading area, (**G**) importin 7 nuclear to cytoplasmic ratio correlated to cell spreading area for control cells and GN-PGCCs cultured on soft and stiff hydrogels. Slopes and *R*^2^ values of linear fitting and *P* values for slope comparisons are shown on the graphs. Statistically significant differences were determined by Student’s *t* test, ***P < 0.001, ****P < 0.0001, *n* = 43, 37, 40, and 37 for Ctrl Soft, Ctrl Stiff, GN-PGCC Soft, GN-PGCC Stiff, respectively. Quantitative data were collected from three independent biological replicates.

**Fig. 6.**
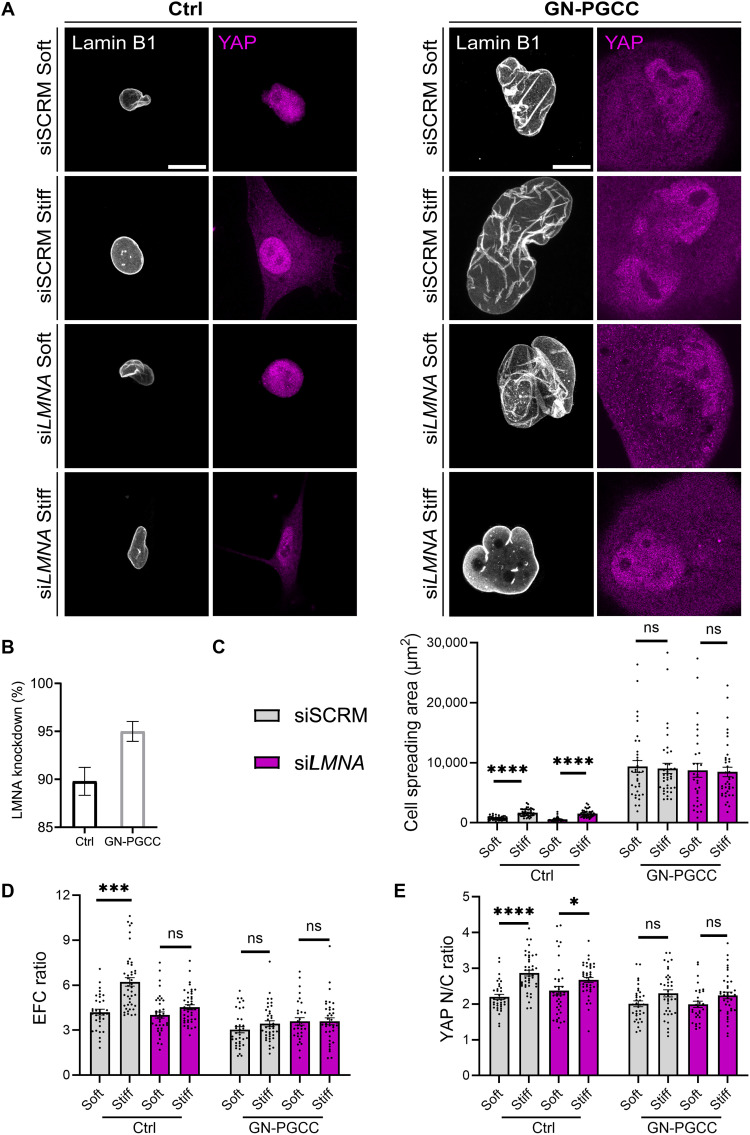
Depletion of *LMNA* does not affect nuclear wrinkling or mechanosensitivity of PGCCs. (**A**) Confocal images of HEY control cells and GN-PGCCs treated with siSCRM or si*LMNA* cultured on soft and stiff polyacrylamide hydrogels stained for Lamin B1 (gray) and YAP (magenta). All scale bars are 20 μm. (**B**) Efficacy of siRNA-mediated knockdown of Lamin A/C in HEY control cells and GN-PGCCs. Expression of lamin A/C in siRNA-treated cells is compared relative to cells treated with scrambled siRNA after normalization of all Ct values to GAPDH expression using the 2^−ΔΔCt^ method. Quantification and comparison of (**C**) cell spreading area, (**D**) EFC ratio, and (**E**) YAP nuclear to cytoplasmic ratio between control cells and GN-PGCCs treated with siRNA and cultured on hydrogels. Statistically significant differences were determined by Student’s *t* test, ***P < 0.001, ****P < 0.0001, *n* = 35, 43, 39, 42, 35, 39, 31, and 39 for siSCRM soft, siSCRM stiff, si*LMNA* soft, si*LMNA* stiff for control cells, and GN-PGCCs, respectively. Quantitative data were collected from three independent biological replicates.

To test this, we synthesized soft and stiff collagen-conjugated polyacrylamide hydrogels (Young’s moduli of 1 kPa, soft, and 308 kPa, stiff, respectively) and cultured HEY GFP–lamin A control cells and GN-PGCCs generated from HEY GFP–lamin A cells on them. As expected, control HEY cells displayed mechanosensitive behaviors. On soft gels, nuclei were wrinkled and cells less spread, while on stiff gels nuclei were smooth and cells were well-spread (Fig. 5A). In contrast, GN-PGCC nuclei were wrinkled on both soft and stiff gels and there was no statistical difference between their EFC ratios (Fig. 5B). GN-PGCCs also failed to spread more on stiff gels (Fig. 5C) and showed no differences in YAP nuclear localization on soft versus stiff gels, unlike control cells (Fig. 5, A and D). Similar results were obtained for importin 7, a nuclear importin required for YAP nuclear import (Fig. 5, A and E) (*56*). Both YAP and importin 7 N/C ratio displayed a clear correlation with the degree of cell spreading in control cells (*R*^2^ = 0.43 and 0.56, respectively), while GN-PGCCs lost this dependence on spreading (*R*^2^ = 0.079 and 0.013, respectively) (Fig. 5, F and G). These results were not substrate specific, as YAP nuclear localization was significantly lower for giant nuclei of spread GN-PGCCs on glass compared to control cells and was similar to GN-PGCCs cultured on stiff gels (fig. S6). We also found that hyperwrinkling persisted even upon siRNA-mediated knockdown of lamin A/C (Fig. 6, A and D), which typically results in higher cell spreading and greater nuclear flattening in noncancer cells (*29*). Consistent with a role for lamin A/C–mediated nuclear tension in mediating YAP translocation, lamin A/C knockdown reduced YAP nuclear to cytoplasmic ratio in nongiant control HEY cells but not in GN-PGCCs (Fig. 6, A and E). Collectively, these findings show that GN-PGCCs have attenuated mechanosensitivity. Unlike their parental cancer cells, they are unable to differentially respond to changes in hydrogel stiffness of two orders of magnitude (1 to 308 kPa) in terms of changes in nuclear unwrinkling, cell spreading or YAP nuclear entry.

GN-PGCCs cultured on even softer hydrogels (Young’s modulus of 0.1 kPa) did display a dramatically lower spreading area compared to the 1-kPa substrate with correspondingly lower YAP N/C ratios (fig. S7). The volume of control and GN-PGCC nuclei was also significantly lower on 0.1-kPa hydrogels compared to 1-kPa hydrogels [volume was not different between 1- and 308-kPa hydrogels consistent with our prior findings in (*32*)]. Control cells did not display large changes in YAP N/C ratio on 0.1-kPa hydrogels compared to 1-kPa hydrogels. These results suggest that mechanosensitivity may not be completely lost in PGCCs. Alternatively, the smaller spreading and lower nuclear volume may reflect a selection mechanism, in which large PGCCs with giant nuclei are unable to adhere to the 0.1-kPa gels.

## DISCUSSION

Reproductive stress, stemming from cancer therapies like chemotherapy or other insults like hypoxia, can drive polyploidization, producing PGCCs with unusually large nuclei (*3*, *4*, *9*, *12*, *36*, *57*). These cells are recognized in tumors by their large size and have been implicated in poor prognosis (*5*, *14*–*16*), therapy resistance (*11*, *12*), and tumor relapse (*58*). While the large size of PGCCs and their nuclei are linked to their abnormal behaviors such as response to chemotherapy drugs (*59*–*61*), PGCC nuclear shape is not well-studied, in part because H&E staining in diagnostic histology does not adequately resolve the nuclear surface (*18*–*23*). Our experiments with immunofluorescence microscopy of lamin B1 in patient tumor tissues show that GN-PGCCs across diverse cancers display hyperwrinkling of the nuclear lamina, a notable feature not detectable in histology (Fig. 1). Our results highlight the usefulness of lamin B1 immunofluorescence imaging in delineating the nuclear periphery with excellent contrast and reveal a previously unrecognized feature of nuclear atypia in cancer.

To determine the cause of hyperwrinkling in GN-PGCCs, we turned to an in vitro model of PGCCs developed from cultured Hey cells (*35*, *36*). Despite some limitations of the model, such as a lack of tissue context and a three-dimensional (3D) microenvironment, the PGCCs displayed giant nuclei and hyperwrinkling behavior like those observed in vivo. Mechanistic experiments with GN-PGCCs ruled out cell rounding (Fig. 2), cytoskeletal indentation or myosin-driven contractility as drivers of laminar hyperwrinkling (Fig. 3). Morphometric measurements show that GN-PGCC nuclei contain threefold more laminar surface area than control nuclei, which even well-spread GN-PGCCs are unable to unfold. Computational modeling confirmed that the measured excess area is much larger than predicted minimum excess area for a smooth nuclear shape (Fig. 4). Thus, hyperwrinkling of the lamina is intrinsic to GN-PGCCs. The origins of this excess laminar area are unclear but likely arise during the laminar assembly step post (failed) mitosis of PGCCs (*32*).

We have recently shown that nuclei in cancer and normal cells conform to the “nuclear drop” model (*30*, *32*, *44*, *62*, *63*). In this model, each nucleus is formed postmitosis with a laminar surface area that is in excess compared to the area of a sphere with the same volume. This results in wrinkles in the lamina of newly formed daughter nuclei (*32*). The presence of these wrinkles allows nuclei to change shape as the daughter cells spread without requiring substantial mechanical energy to deform the nucleus. Complete unwrinkling of the nuclear lamina that typically occurs in cultured, spread cells is responsible for the nuclei reaching a steady-state shape (*32*), because it results in a taut, smooth lamina that is inextensible to cellular forces (*26*, *30*, *32*, *64*, *65*). The presence of the large number of wrinkles in GN-PGCC nuclei, which prevents the development of in-plane tension, may explain how these nuclei become even more flattened than control nuclei, which possess a taut lamina. In hyperwrinkled nuclei, it is possible that the nucleus reaches a steady state shape during flattening owing to the many folds in the lamina that may become trapped during the downward compression of the nucleus.

We have previously proposed that the development of surface tension by unwrinkling of the nuclear lamina can promote the nuclear localization of YAP (*32*). The presence of lamin A/C is required for the development of surface tension in the unwrinkled lamina (*26*, *63*) and depletion of lamin A/C reduces YAP nuclear localization (*32*). Consistent with this, we found that YAP localization was higher in the smooth nuclei in nongiant control HEY cells cultured on stiff ECM compared to the wrinkled nucleus in control cells cultured on soft ECM. The hyperwrinkling in giant nuclei likely prevents the development of in-plane tension in the lamina and YAP remains cytoplasmic in these cells as a result. The mechanisms by which laminar tension promotes YAP nuclear localization are not known. An absence of tension in the lamina may promote phosphorylation of YAP by the Hippo pathway kinases through as yet unknown mechanisms, preventing YAP binding to importin 7, and leading to YAP retention in the cytoplasm (*56*).

Tumor stiffening that accompanies tumor progression is known to promote cell proliferation (*66*–*68*), and such mechanosensitive proliferation is mediated by YAP nuclear localization (*67*–*69*). Therefore, the absence of mechanosensitive YAP nuclear localization in GN-PGCCs may account for their relative quiescence (*70*–*72*) that precedes the rare catastrophic division event into many daughter cells (*3*, *12*, *13*).

Our data collectively establish nuclear hyperwrinkling as an intrinsic feature of GN-PGCCs and as a novel type of nuclear atypia in tumors. This laminar hyperwrinkling may disrupt mechanobiological signaling pathways that normally couple mechanical cues to tumor cell proliferation. Further studies are needed to define the molecular basis of excess laminar area in giant nuclei and to understand the full implications of hyperwrinkling for PGCC dysfunction.

## MATERIALS AND METHODS

### Cell culture

Cell lines were maintained in a humidified incubator at 37°C and 5% CO_2_. Human ovarian cancer cells HEY (Cytion, 305017) were cultured in Dulbecco’s modified Eagle’s medium with glucose (4.5 g/liter; Corning), supplemented with 10% (v/v) fetal bovine serum (Gibco) and 1% (v/v) penicillin/streptomycin (Corning). GN-PGCCs were formed by treating HEY WT and GFP–lamin A cells with 10 nM docetaxel for 2 periods of 72 hours, separated by 24 hours in fresh culture medium. To culture cells on glass-bottom dishes, the surface was coated with human fibronectin (0.1 μg/ml; Corning) in deionized water at room temperature for 30 min, then washed with phosphate-buffered saline (PBS). After seeding, cells were allowed to spread for 3 hours for control cells and 24 hours for PGCCs before fixation. GFP-fusion lamin A was stably expressed in cancer cell lines by retroviral transduction as previously described (*32*).

### Immunofluorescence staining and microscopy

Cells cultured on glass or hydrogels were fixed in 4% paraformaldehyde (PFA; Thermo Fisher Scientific) for 15 min at room temperature, permeabilized with 0.1% Triton X-100 (Thermo Fisher Scientific) in PBS for 30 min, and blocked with SuperBlock blocking buffer (Thermo Fisher Scientific) for 1 hour at room temperature. Cells were then incubated with primary antibodies rabbit anti–lamin B1 (Abcam, ab229025; dilution 1:1000), rabbit anti–ɑ-tubulin (Abcam, ab18251; dilution 1:2000), and mouse anti-YAP (Santa Cruz Biotechnology, sc-101199; dilution 1:100) diluted in SuperBlock overnight at 4°C. The sample was washed with PBS thrice and incubated with secondary antibodies Alexa Fluor 594 goat anti-rabbit (Invitrogen, dilution 1:200), Alexa Fluor 594 goat anti-mouse (Invitrogen, dilution 1:200), or Alexa Fluor 647 goat anti-mouse (Invitrogen, dilution 1:200) diluted in PBS for 2 hours at room temperature. DNA and F-actin were stained with Hoechst 33342 (Sigma-Aldrich) and phalloidin conjugated to Alexa Fluor 405 or 488 (Invitrogen; dilution 1:400), respectively.

For importin 7 staining, cells were washed three times in 37°C PBS and fixed in 4% PFA for 15 min at 37°C (*56*). The cells were then washed with PBS and permeabilized with 0.1% Triton X-100 for 10 min at room temperature and incubated with primary antibody rabbit anti-importin 7 (Proteintech, dilution 1:200) for 1 hour at room temperature. The cells were washed with PBS and incubated in secondary antibody Alexa Fluor 594 goat anti-rabbit (1:200) for 1 hour at room temperature.

Live and fixed imaging was conducted using 60×/1.3 numerical aperture (NA) or 60×/1.5 NA oil-immersion objectives on an Olympus FV3000 confocal microscope (Olympus Scientific Solutions Americas Corp.). Brightness and contrast were adjusted for some images for visualization. A *z*-step size of 130 nm and a pinhole size of 1 Airy disk were used for 3D confocal imaging to sample at less than half the focus depth to satisfy the Nyquist criterion

$$\begin{array}{c} \frac{\text{FWHM}}{2}=\frac{1}{2}\cdot \frac{0.88\cdot \lambda_{\text{ex}}}{n-\sqrt{n^{2}-{\text{NA}}^{2}}}=\frac{1}{2}\cdot \frac{0.88\cdot 488\;\text{nm}}{1.5-\sqrt{1.5^{2}-1.4^{2}}}\\ \approx 223\;\text{nm}>130\;\text{nm} \end{array}$$ (1)

where FWHM is the full width at half maximum, $\lambda_{\text{ex}}$ is the excitation wavelength, n is the refractive index of the immersion medium, and NA is the lens NA.

Super-resolution imaging was performed on a Nikon N-STORM microscope using a 60×/1.42 NA silicone oil-immersion objective. A *z*-step size of 128 nm was used.

FFPE tissues were immunostained and imaged as described previously (*19*). Some images used here were collected from this previous study. In brief, tissues were deparaffinized, treated for antigen retrieval in an instant pot on high for 20 min with a 1× universal antigen retrieval solution (Abcam), blocked, and immunostained overnight at 4°C for lamin B1 (Abcam, diluted 1:2000) and pan-cytokeratin (LS-Bio, diluted 1:50). Secondary staining was performed at 1:500 dilution for 1 hour at room temperature, followed by imaging on an Olympus confocal microscope (FV3000) with a 60× (NA = 1.50) objective or a 60× (NA = 1.30) objective. Brightness and contrast were adjusted for some images for visualization. GN-PGCCs were identified from previously collected images as nuclei in focus appearing around 3× the size of at least some of their neighboring nuclei.

H&E staining was performed as described previously (*19*) on the same tissue arrays that were previously immunostained for lamin and pan-cytokeratin. H&E images were collected with a color camera (Olympus DP23) on the Olympus microscope using 60× objectives (NA = 1.3 and 1.5) or with a 63× (NA = 1.4) objective on a Leica DM 6B upright microscope.

### Synthesis and functionalization of polyacrylamide hydrogels

Polyacrylamide hydrogels were synthesized as previously described (*32*). Acrylamide and bis-acrylamide (Bio-Rad Laboratories) were combined at 5/0.1% and 15/1.2% to prepare gels with Young’s modulus (*E*) of 1 and 308 kPa, respectively. The precursor solution was desiccated, then mixed with 0.5% (v/v) ammonium persulfate (Thermo Fisher Scientific) and 0.1% (v/v) tetramethylethylenediamine (Thermo Fisher Scientific) to initiate polymerization, and 25 μl of the solution was layered between a hydrophobic 18-mm glass coverslip and a hydrophilic glass-bottom 35-mm dish (World Precision Instruments) at room temperature for 20 min. The gels were functionalized using sulfosuccinimidyl 6-(40-azido-20-nitophenylamino) hexanoate (Sulfo-SANPAH, G-Biosciences) and coated with rat tail collagen type I (0.2 mg/ml; Corning) overnight. Hydrogels were washed with PBS and incubated at 37°C before cell seeding.

### Transfection of siRNAs

Transfection of cells was carried out as previously described (*30*). HEY WT control cells and PGCCs were cultured in 12-well plates and 25-cm^2^ culture flasks, respectively, in an antibiotic-free medium at 40% confluency at the time of transfection. The transfection solution consisted of 0.25% lipofectamine RNAiMAX transfection reagent (Invitrogen) and 0.4% siRNA for control cells and 0.8% siRNA for PGCCs (Dharmacon, siGENOME Non-Targeting Control siRNA Pool no. 2, D-001206-14-05, target sequences: UAAGGCUAUGAAGAGA UAC, AUGUAUUGGCCUGUAUUAG, AUGAACGUGAAUUGCUCAA, and UG GUUUACAUGUCGACUAA; *LMNA* siGENOME SMARTpool siRNA, D004978-01, target sequence: GAAGGAGGGUGACCUGAUA; IPO7 siGenome SMARTpool siRNA, D-012255, target sequences: GAAGAUCGCCAUUGUAUUC, GGAAUCUGCUUACAGGUCA, GUAUUGGCCUGAUCGAGAA, and GCACUGACUCACGGUCUUA) in the reduced serum Opti-MEM medium (Gibco). The culture medium was replaced with transfection solutions, and cells were incubated in 5% CO_2_ for 3 days. After 48 hours for control cells and 72 hours for PGCCs, cells were collected for polymerase chain reaction (PCR) assay to verify siRNA knockdown and were passaged onto polyacrylamide hydrogels.

### Reverse transcriptase-quantitative PCR

To quantify the efficacy of the siRNA knockdown, cells were lysed and purified using the RNeasy Plus Kit (Qiagen), then RNA concentration was determined. RNA, reverse transcriptase, oligo primers, and nucleotide triphosphates (dNTPs) (iScript Advanced cDNA Synthesis Kit, Bio-Rad Laboratories) were mixed in PCR-grade nuclease-free water (Invitrogen) to generate cDNA. The reaction mixture was incubated in the Bio-Rad thermocycler at the appropriate temperature and duration for reverse transcription. The PCR reaction combined cDNA, primers, DNA polymerase, dNTPs, and iQ SYBR Green Supermix reaction buffer (Bio-Rad Laboratories). Forward and reverse primer sets [GAPD: VHPS-3541, forward (5′-3′): GAGTCAACGGATTTGGTCGT, reverse (5′-3′): TTGATTTTGGAGGGATCTCG, RealTimePrimers; *LMNA*: Ref# 460260112 & 13, forward sequence: ATGAGGACCAGGTGGAGCAGTA, reverse sequence: ACCAGGTTGCTGTTCCTC-TCAG] were used to target the cDNA of the genes of interest. PCR amplification was performed using appropriate cycling conditions recommended by Bio-Rad. The standard curves and Ct values were used to quantify gene expression levels for the genes of interest normalized to the expression level of GAPD in the treatment group relative to the normalized gene expression levels in the siSCRM group.

### Image analysis

We used a customized MATLAB code to analyze nuclear morphometry as previously described in detail (*32*). In brief, an Otsu segmentation algorithm was used to generate and segment the maximum-projection images of the lamin channel. Nuclear masks were created using these maximum-projection images for the identification of nuclei, and nuclei were individually cropped by applying the resulting nuclear masks to lamin *z*-stack images. Nuclear height was quantified by calculating mean lamin intensity across *z*-planes and identifying the bottom and top confocal planes as the points where intensity rose above and fell below background. Height was measured as the distance between these planes. The MATLAB Image Processing Toolbox was used to quantify nuclear surface area and volume. Nuclear aspect ratio was quantified by measuring the major axis using the *x*-*y* view and dividing by the nuclear height as calculated previously. We captured and quantified nuclear irregularities using an elliptical Fourier analysis, as reported previously (*40*). This method approximates nuclear shapes through the decomposition of the shape into a series of harmonic ellipses (*41*). We improved the Otsu segmentation by tracing the maximum intensity on each normal line along the nuclear periphery to delineate the precise nuclear contour at subpixel resolution. The precise nuclear contour was accurately approximated using a series of elliptic harmonics derived from the Fourier analysis coefficients of the *x* and *y* coordinates of the nuclear outline (*31*, *40*). Shape irregularity was quantified using the EFC ratio, calculated by comparing the combined lengths of the major and minor semiaxes at the first harmonic to those of the next 14 higher-frequency harmonics. A smooth, regular nuclear contour, well represented by the first harmonic with minimal contributions from higher frequencies, leads to a higher EFC ratio. Conversely, a more irregular contour requires greater input from higher-frequency harmonics, leading to a lower EFC ratio. To account for variations of nuclear irregularity across different confocal planes, we extracted contours at 75, 50, and 25% of the nuclear height, representing the top, middle, and bottom, and averaged their EFC ratios of three planes for each nucleus. Cell masks were generated by applying Otsu segmentation to maximum-projection images of the phalloidin channel. Cell spreading area was quantified using MATLAB Image Processing Toolbox. The nuclear-to-cytoplasmic YAP and importin 7 ratios were calculated by applying nuclear and cell masks to maximum-projection YAP and importin 7 images and using the formula: [(Nuclear intensity) − (Background intensity)]/[(Cytoplasmic intensity) − (Background intensity)].

### Nuclear classification using deep learning model

The full versions of the cropped images of tissues shown in Fig. 1 were cropped into individual nuclei and passed through our established deep learning model to sort nuclei into different classes of nuclear wrinkling (shown in fig. S3A), as described previously (*19*). The results from this analysis were reported as the pie chart for “all nuclei” in fig. S3B. As PGCC were not always cropped properly by our method, all PGCC in Fig. 1 were cropped by hand and passed through the deep learning model. In order to generate the “all nuclei” column in table S1, all tissue images checked for PGCC were cropped into individual nuclei.

### Cytoskeleton and contractility disruption

For the cytoskeleton disruption experiments, GFP–lamin A–expressing HEY PGCCs were cultured on fibronectin-coated glass-bottom dishes and treated with 50 μM cytochalasin D (Sigma-Aldrich) or nocodazole (STEMCELL Technologies) for 2 hours. Z-stack live imaging was performed both before and after treatment with a 130-nm step size to evaluate nuclear wrinkling. After treatment, cells were fixed and stained for F-actin and microtubules to verify disruption. For the contractility disruption experiments, GFP–lamin A–expressing HEY PGCCs were cultured on fibronectin-coated glass-bottom dishes and treated with 50 μM blebbistatin (Sigma-Aldrich) or Y-27632 (Sigma-Aldrich) for 3 hours. Z-stack live imaging was performed both before and after treatment with a 130-nm step size to evaluate nuclear wrinkling. After treatment, cells were fixed and stained for F-actin. Live-imaging z-stacks of lamin A before and after treatment were used to determine the wrinkling state of each nucleus.

### Determination of minimum nuclear surface area confined by the cell cortex

As demonstrated previously (*32*, *44*), the cortical and nuclear surface of fully spread cells tend to be surfaces of constant mean curvature (fig. S8). The cell and nuclear shapes of a fully spread, axisymmetric cell can be calculated for given spread area, cell volume, nuclear volume, and nuclear surface area as follows. In general, an axisymmetric surface of constant mean curvature is characterized by radius R(ϕ) versus vertical position z(ϕ), parameterized by variable ϕ, with constant mean curvature, *H*. The equations for R(ϕ) and z(ϕ) are

$$R\left(\phi \right)=\sqrt{\alpha^{2}{\text{cos}}^{2}\phi +\beta^{2}{\text{sin}}^{2}\phi }$$ (2)

z(ϕ)=αE(ϕ,k)+βF(ϕ,k) (3)

where F(ϕ,k) and E(ϕ,k) are incomplete elliptical integrals of the first and second kinds, respectively, and

$$\alpha =\frac{1+\sqrt{1-4\mathrm{CH}}}{2H}\beta =\frac{1-\sqrt{1-4\mathrm{CH}}}{2H}$$

Here, *C* is parameter that establishes where the surface is a nodoid or unduloid surface of rotation [see Dickinson and Lele (*44*) for a detailed mathematical derivation]. As illustrated in fig. S8, the axisymmetric cell and nucleus shapes are comprised of the nucleus-cortex interface (a spherical cap with mean curvature $H_{\text{cap}}$ and parameter $C_{\text{cell}}=0$), the nucleus-cytoplasm interface (a nodoid with mean curvature $H_{\text{nuc}}$ and $C_{\text{nuc}}>0$), and the cortical surface away from the nucleus (either nodoid or unduloid with mean curvature $H_{\text{cell}}$ and $C_{\text{cell}}$ of either sign depending on the spread area). Defining ($R_{1},z_{1}$) as the junction point of the three surfaces and equating dR/dz at this point yields

$$C_{\text{cell}}=\left(H_{\text{cap}}-H_{\text{cell}}\right){R_{1}}^{2}\;C_{\text{nuc}}=\left(H_{\text{cap}}-H_{\text{nuc}}\right){R_{1}}^{2}$$ (4)

The radius $R_{n0}$ where the nucleus surface impinges tangentially (dz/dR = 0) on the substratum, is given by

$$R_{n0}=R_{1}\sqrt{1-\frac{H_{\text{cap}}}{H_{\text{nuc}}}}$$ (5)

For a given cell spread area, the specific cell and nuclear shapes can then be calculated by solving for the values of $H_{\text{cap}}$, $H_{\text{cell}}$, and $H_{\text{nuc}}$ that yield specified values of cell volume, nuclear volume, and nuclear surface area.

In the present study, the cell shapes of approximately axisymmetric cells were taken as the *x*-*z* cross sections of the cell cortex. A hypothetical nuclear surface with minimum surface area was found by varying the nuclear surface area to fit the calculated *x*-*z* profile to the observed cell shape for a given (measured) nuclear volume. The resulting calculated nuclear shape is that of the smallest surface area (for the measured nuclear volume) that can be confined beneath the cell cortex.

### Statistical analysis

All quantitative data are presented as mean ± SEM. GraphPad Prism 10.0 was used for statistical analysis and graphic representations of data. The following symbols were used to denote significance: NS: *P* > 0.05, *P ≤ 0.05, **P ≤ 0.01, ***P ≤ 0.001, and ****P ≤ 0.0001. The details of experimental conditions and statistical tests are provided in the figure legends.
