# Supplementary material for: Hyperwrinkling of the nuclear lamina is associated with attenuated mechanosensitivity in giant nucleated cancer cells
Source: Sci Adv. 2026 Jul 17;12(29):eaed1645. doi: 10.1126/sciadv.aed1645 (PMC13378562; doi:10.1126/sciadv.aed1645)
Supplement: Supplementary file 1 — Figs. S1 to S8 Table S1 [file sciadv.aed1645_sm.pdf]

Science Advances
AAAS

Supplementary Materials for

Hyperwrinkling of the nuclear lamina is associated with attenuated mechanosensitivity in giant nucleated cancer cells

Samere Abolghasemzade *et al.*

Corresponding author: Tanmay P. Lele, tanmay.lele@tamu.edu

Sci. Adv. **12**, eaed1645 (2026)
DOI: 10.1126/sciadv.aed1645

This PDF file includes:

Figs. S1 to S8
Table S1

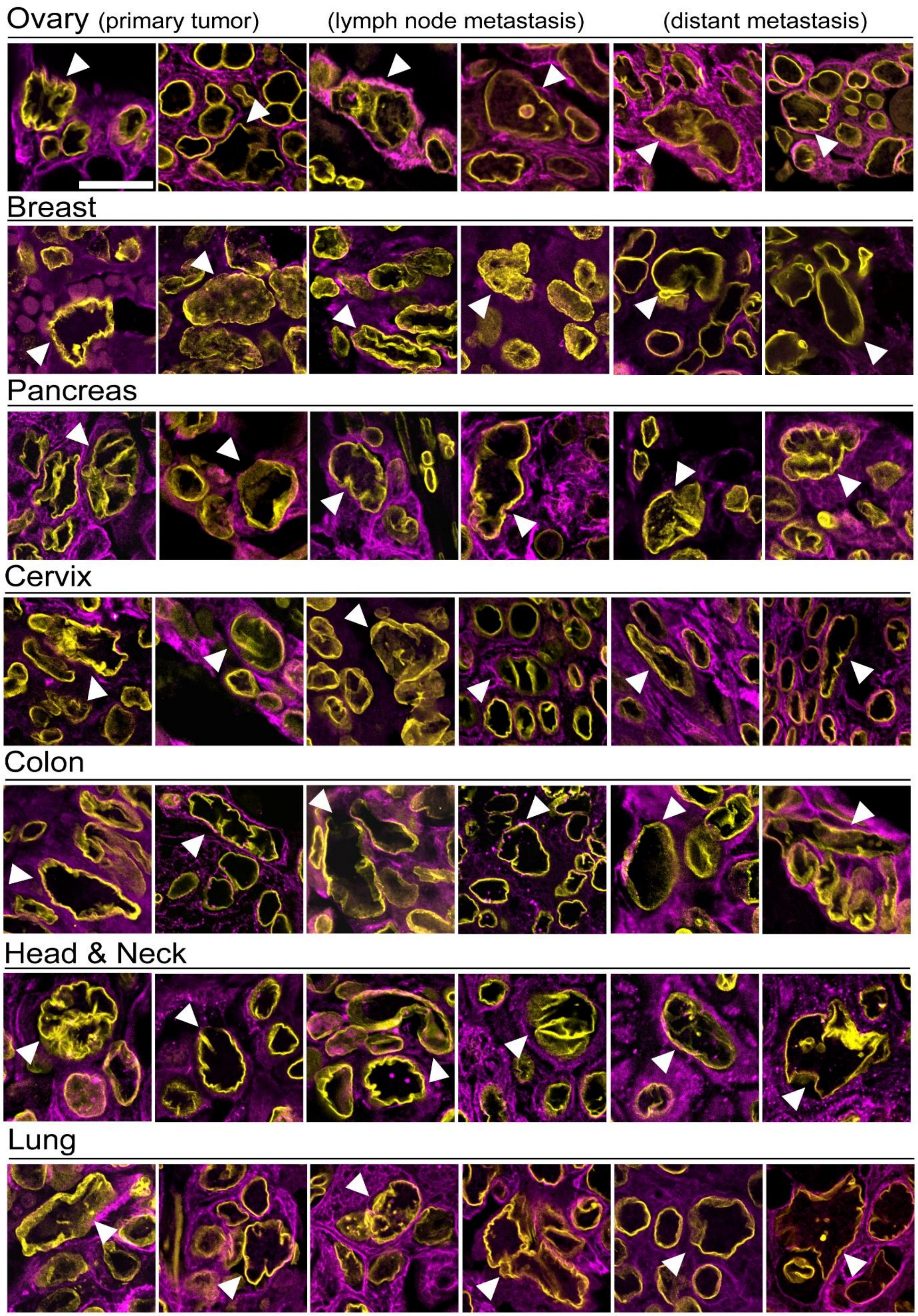

Ovary (primary tumor)
(lymph node metastasis)
(distant metastasis)
Breast
Pancreas
Cervix
Colon
Head & Neck
Lung

Supplementary Figure 1: Additional giant nuclei in GN-PGCCs are represented in diverse tumor tissues.

Zoomed regions of 60x confocal images of giant nuclei in FFPE tissue stained for lamin B1 (yellow) and pan-cytokeratin (magenta) in 60x ovarian (primary and metastatic), and primary tumors of breast, pancreatic, cervical, colon, head and neck, and lung cancer. Location of giant nuclei are marked by white arrows. Regions were selected by the presence of a nucleus at least three times as large as surrounding epithelial cells. All scale bars are 20 μm.

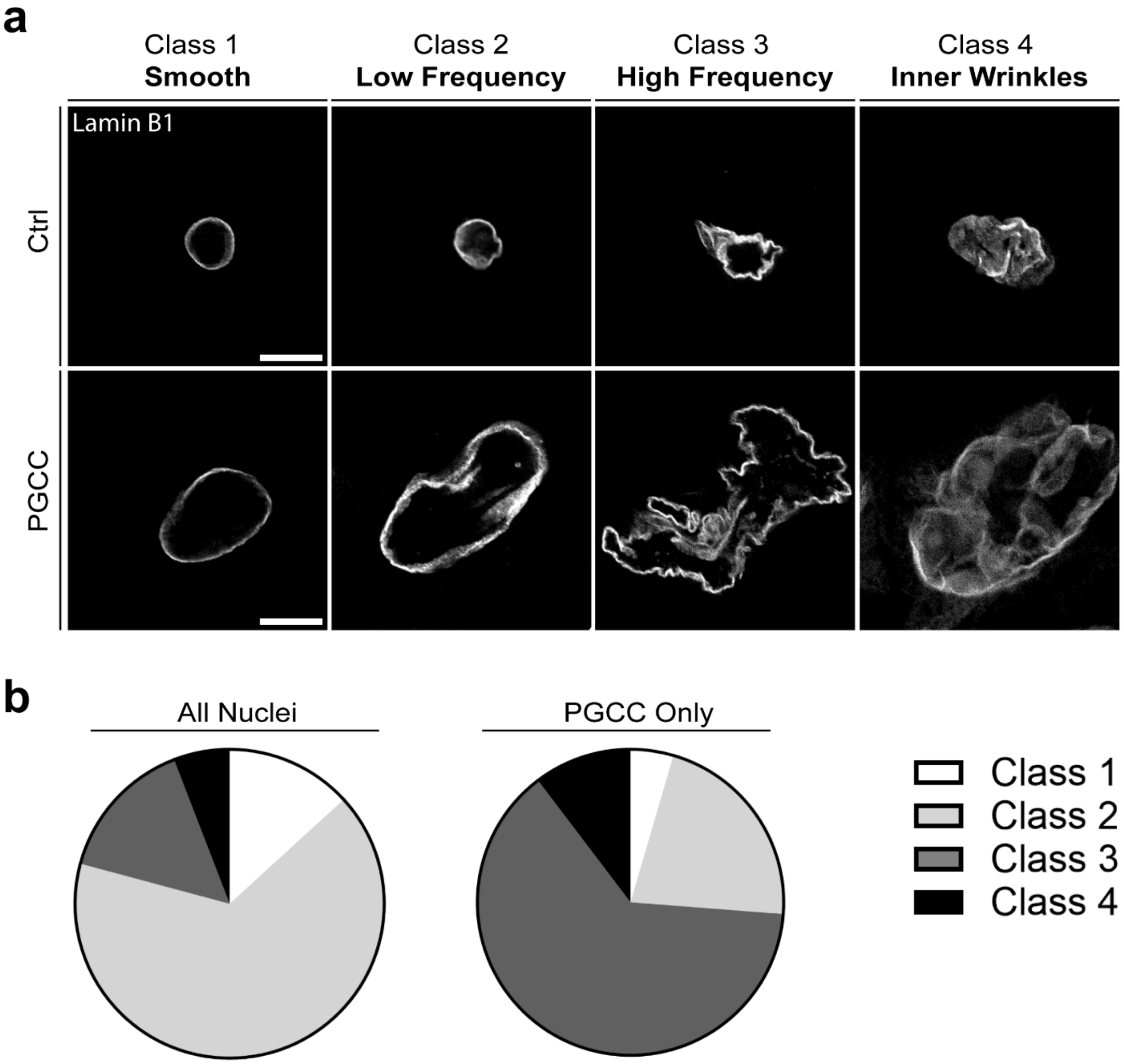

Supplementary Figure 2: Extreme wrinkling, detected by deep learning, is more prevalent in giant nuclei.

a) Examples of representative nuclei sorted into classes 1-4 using a deep learning model based on nuclear shape. 1 = smooth, 2 = low frequency, 3 = high frequency, 4 = inner wrinkles. Scale bar is 10 μm **b)** Pie charts showing the normalized corrected counts of nuclei in each class for the total population and for PGCCs. n=115, 115 for all nuclei and PGCCs, respectively. Surrounding nuclei were removed for improved visualization.

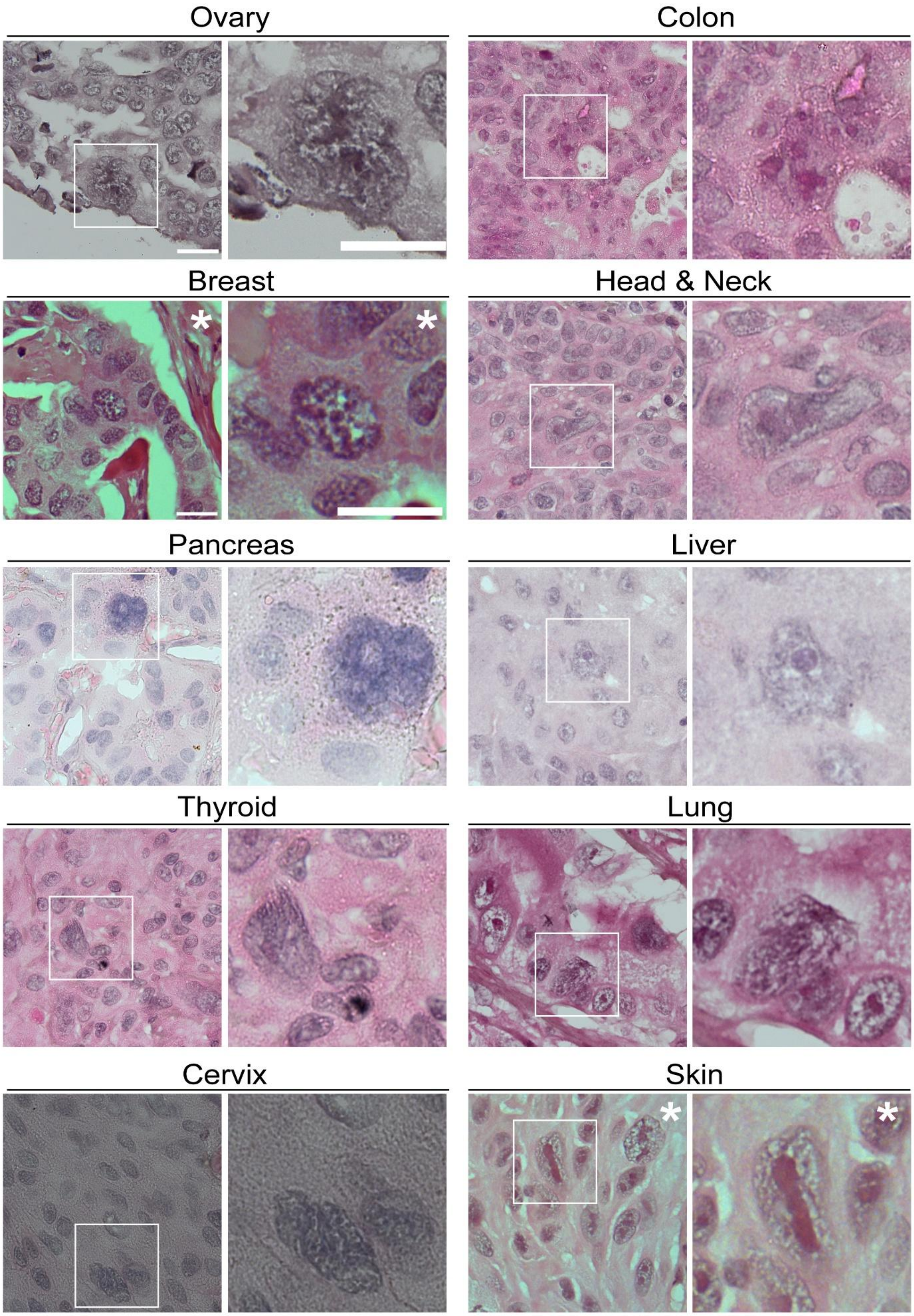
Ovary
Colon
Breast
Head & Neck
Pancreas
Liver
Thyroid
Lung
Cervix
Skin

Supplementary Figure 3: H&E staining does not visualize fine contours of the giant nucleus.

Brightfield images of giant nuclei in H&E-stained cancer tissues taken using 60x objectives corresponding to the same regions in immunostained tissue, whose images are shown in **Fig. 1**. All scale bars are 20 μm. Images with white asterisks were taken using 63x objective. Zoomed regions denoted by the white box are also shown.

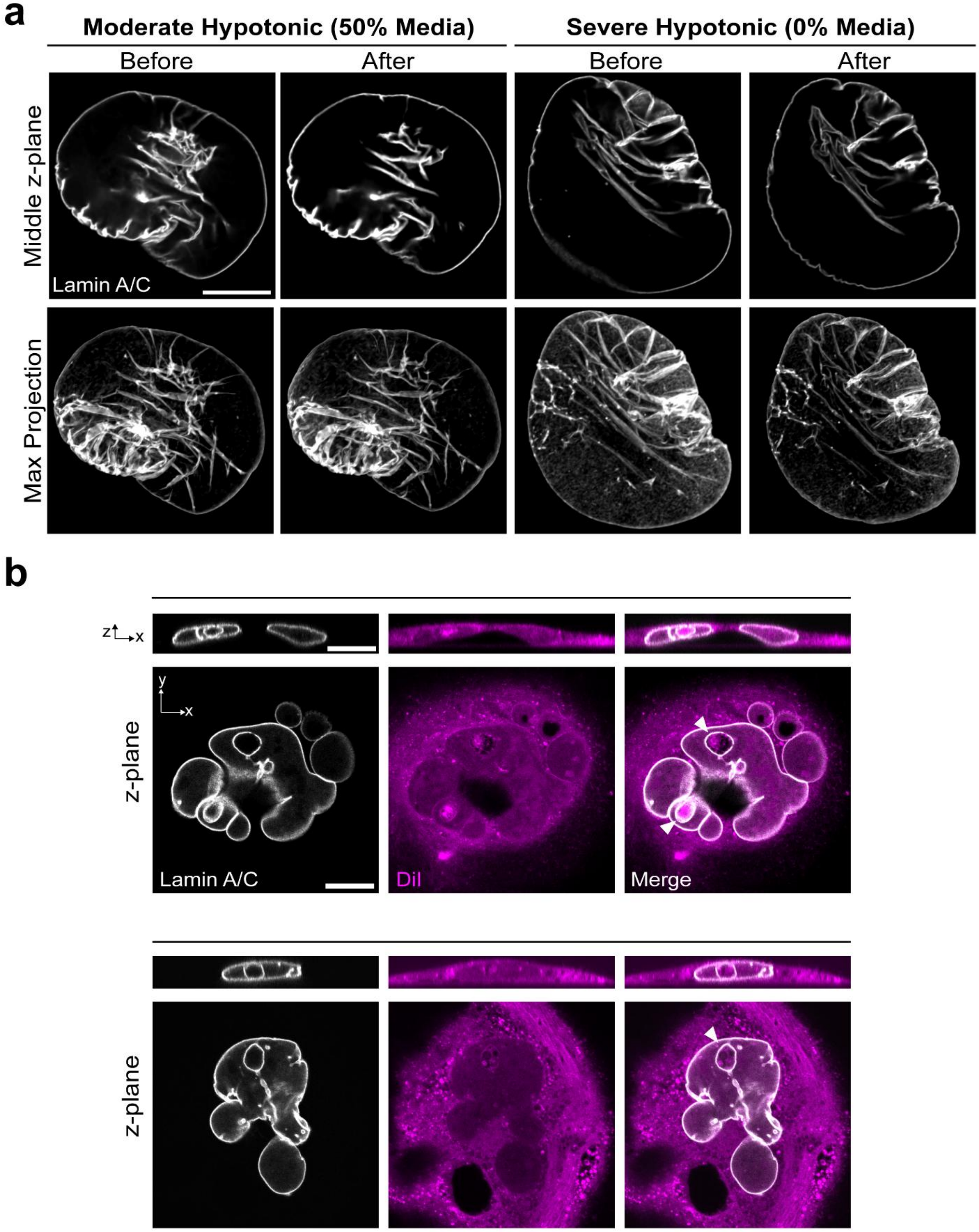

a
Moderate Hypotonic (50% Media)
Severe Hypotonic (0% Media)
Before
After
Before
After
Middle z-plane
Max Projection
Lamin A/C
b
z
x
y
x
z-plane
Lamin A/C
DiI
Merge
z-plane

Supplementary Figure 4

Confocal images of GN-PGCCs expressing GFP-Lamin A (gray) cultured on fibronectin-coated glass. **a)** Confocal live images of GN-PGCC cells expressing GFP-LMNA (grey) before and 5 minutes after moderate (50% media) and severe (0% media) hypo-osmotic shock. **b)** Confocal fixed images of GN-PGCCs stained with DiI (magenta). Location of lipid-containing holes denoted with white arrows. All scale bars are 20 μm.

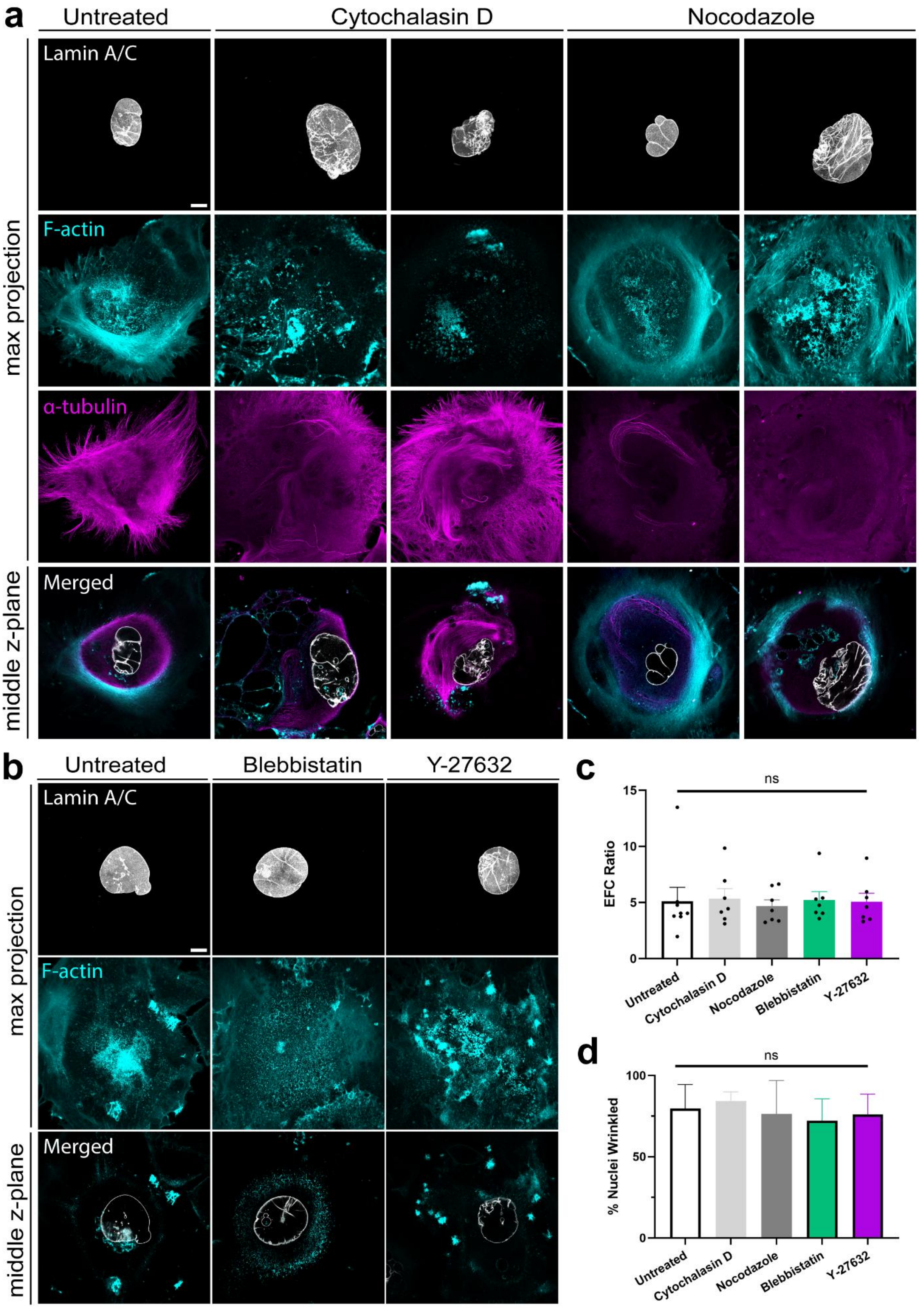
a
Untreated
Cytochalasin D
Nocodazole
Lamin A/C
F-actin
α-tubulin
Merged
max projection
middle z-plane
b
Untreated
Blebbistatin
Y-27632
Lamin A/C
F-actin
Merged
max projection
middle z-plane
c
ns
EFC Ratio
15
10
5
0
Untreated
Cytochalasin D
Nocodazole
Blebbistatin
Y-27632
d
ns
% Nuclei Wrinkled
100
50
0
Untreated
Cytochalasin D
Nocodazole
Blebbistatin
Y-27632

Supplementary Figure 5: Effect of the disruption of cytoskeletal forces on nuclear wrinkling in GN-PGCCs.

Effect of pharmacological drug treatment on laminar hyperwrinkling in GN-PGCCs. a) and b) show confocal max projection and middle z-plane images of fixed GN-PGCCs expressing GFP-Lamin A (grey) treated with **a)** cytochalasin D, and nocodazole, **b)** blebbistatin, and Y-27632 and stained for F-actin (cyan) and α-tubulin (magenta). Untreated GFP-lamin A expressing GN-PGCCs were used as control. All scale bars are 20 μm. **c)** Quantification of EFC ratio for fixed GN-PGCCs expressing GFP-lamin A under the various drug treatments; n = 8 and 7 for control and each drug-treated group, respectively, **d)** Comparison of percentage of wrinkled nuclei in fixed WT GN-PGCCs (i.e. not expressing GFP lamin A) under the different drug treatments; n = 24 for each group. All data in this figure was collected from three independent biological replicates.

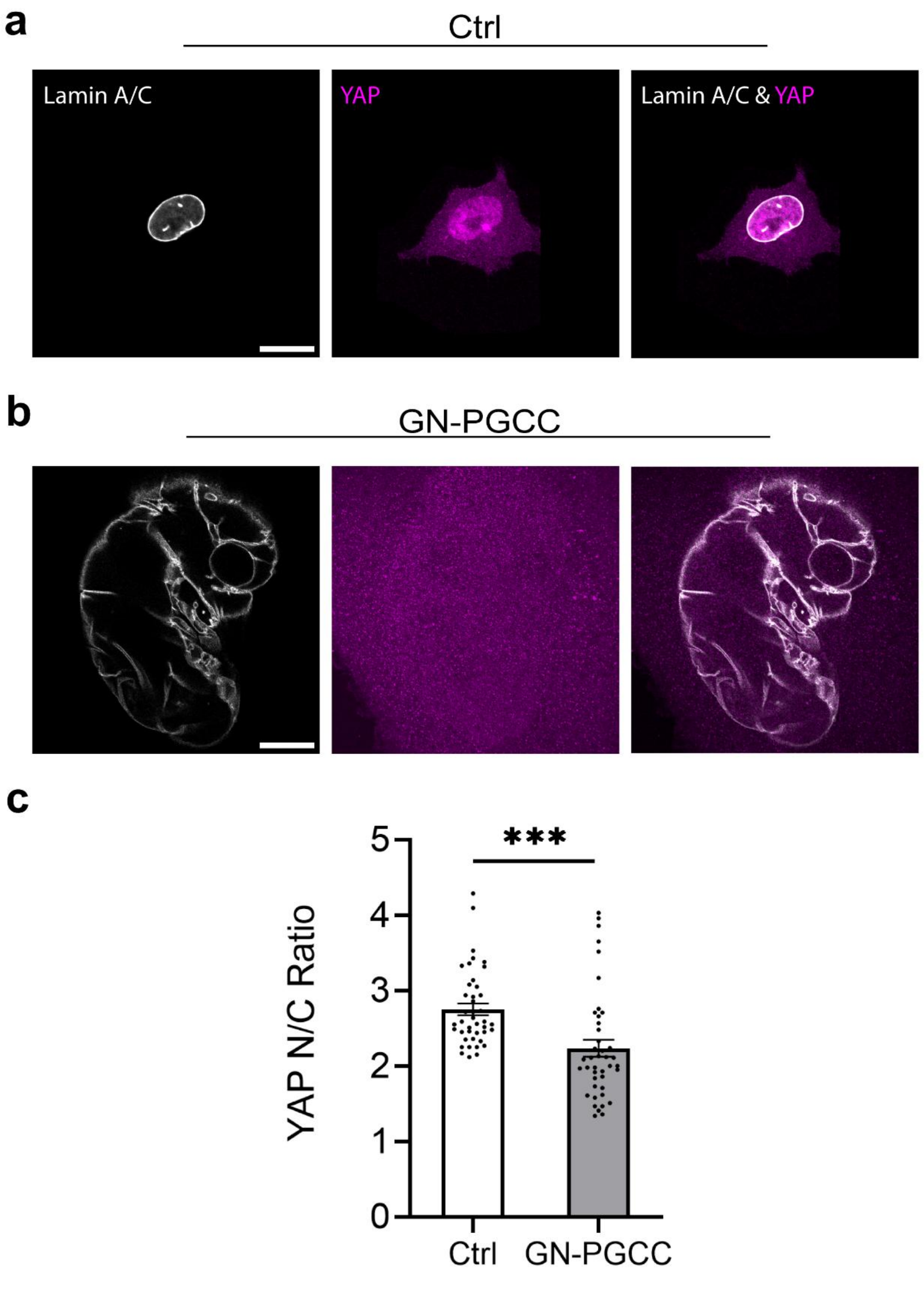
a
Ctrl
Lamin A/C
YAP
Lamin A/C & YAP
b
GN-PGCC
c
YAP N/C Ratio
***
0
1
2
3
4
5
Ctrl
GN-PGCC

Supplementary Figure 6: YAP nuclear localization is significantly lower in GN-PGCCs than parental cancer cells on glass.

Confocal images of **a)** HEY control and **b)** GN-PGCC cells expressing GFP-Lamin A (gray) cultured on glass. Cells were stained YAP (magenta). All scale bars are 20 μm. **c)** Quantification and comparison of YAP nuclear to cytoplasmic ratio between control cells and GN-PGCCs. ***$p < 0.001$, ****$p < 0.0001$, n= 42, 42 for control cells and GN-PGCCs, respectively. Quantitative data was collected from three independent biological replicates.

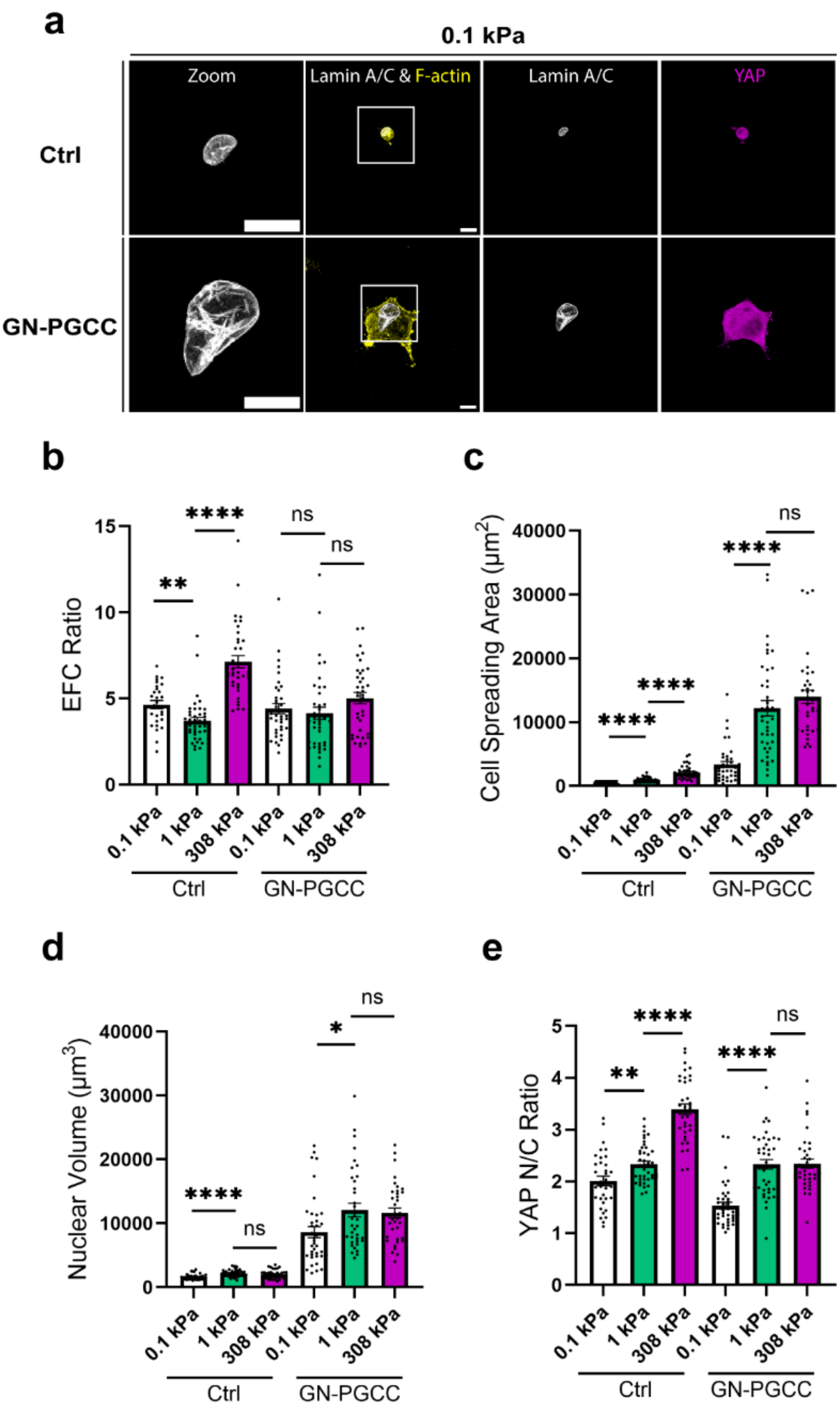

a
0.1 kPa
Zoom
Lamin A/C & F-actin
Lamin A/C
YAP
Ctrl
GN-PGCC
b
EFC Ratio
c
Cell Spreading Area (μm²)
d
Nuclear Volume (μm³)
e
YAP N/C Ratio
0.1 kPa
1 kPa
308 kPa
Ctrl
GN-PGCC
ns

Supplementary Figure 7: GN-PGCC culture on 0.1 kPa hydrogels.

a) Confocal images of HEY control cells and GN-PGCCs expressing GFP-Lamin A (grey) cultured on very soft (0.1kPa) polyacrylamide hydrogels. Cells were stained for F-actin (yellow), and YAP (magenta). All scale bars are 20 μm. Quantification and comparison of **b)** EFC ratio, **c)** cell spreading area, **d)** nuclear volume, and **e)** YAP nuclear to cytoplasmic ratio. Statistically significant differences were determined by student's t-test, ***$p < 0.001$, ****$p < 0.0001$, $n = 34$, 43, 37, 37, 40, 37 for Ctrl 0.1 kPa, Ctrl 1 kPa, Ctrl 308 kPa, GN-PGCC 0.1 kPa, GN-PGCC 1 kPa, GN-PGCC 308 kPa, respectively. Quantitative data was collected from three independent biological replicates.

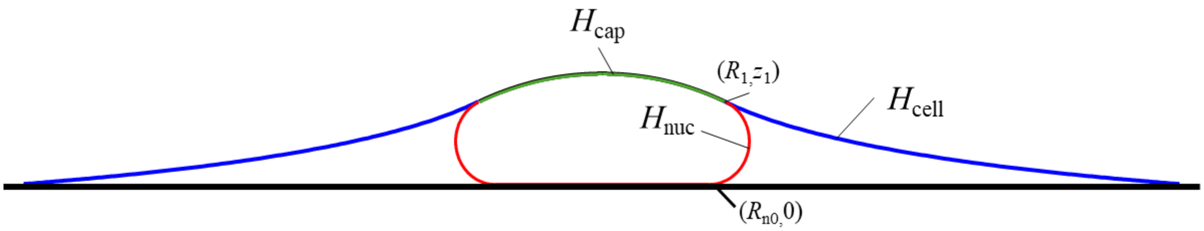

Supplementary Figure 8

The characteristic cell and nuclear shapes of an axisymmetric, fully spread cell consist of three surfaces of constant mean curvature: the nucleus-cytoplasm interface (red), a nodoid of constant mean curvature H_{nuc}, the nucleus-cortex surface (green), a spherical cap of constant mean curvature H_{cap}; and the cortical surface away from the nucleus (blue), a nodoid or unduloid surface of constant mean curvature H_{cell}. The three surfaces intersect at point (R_1, z_1) and the nucleus contacts the substratum (black) at radius, R_{n0}.

**Ovary**	Normal / Normal Adjacent	Primary Tumors	Metastatic Tumors
Number of PGCC Nuclei	0	17	9
All Nuclei	197	68320	7879
**Breast**	Cancer Adjacent	Primary Tumors	
Number of PGCC Nuclei	1	11	
All Nuclei	12109	24366	
**Pancreas**	Normal	Primary Tumors	
Number of PGCC Nuclei	1	9	
All Nuclei	1557	4516	
**Thyroid**	Normal	Primary Tumors	
Number of PGCC Nuclei	0	2	
All Nuclei	670	16323	
**Cervix**	Normal	Primary Tumors	
Number of PGCC Nuclei	0	10	
All Nuclei	828	9573	
**Colon**	Normal	Primary Tumors	
Number of PGCC Nuclei	3	14	
All Nuclei	6602	44264	
**Head and Neck**	Normal / Normal Adjacent	Primary Tumors	
Number of PGCC Nuclei	0	10	
All Nuclei	6075	21638	
**Liver**	Normal	Primary Tumors	
Number of PGCC Nuclei	0	6	
All Nuclei	644	5860	
**Lung**	Normal	Primary Tumors	
Number of PGCC Nuclei	0	11	
All Nuclei	1546	15420	
**Skin**	Cancer Adjacent	Primary Tumors	
Number of PGCC Nuclei	0	11	
All Nuclei	2334	23114	

Supplementary Table 1

The number of PGCC nuclei and total nuclei in normal or cancer adjacent tissue, primary tumors, and metastatic tumors in ten different carcinomas, counted from lamin B1 and pan-cytokeratin-immunostained sections. Cells were considered to be epithelial if they expressed pan-cytokeratin.
